# Thoracic approach for suspected internal mammary lymph node recurrence following mastectomy and implant-based reconstruction: a case report

**DOI:** 10.1093/jscr/rjae829

**Published:** 2025-01-04

**Authors:** Daisuke Murayama, Ryosuke Hirano, Osamu Mishima, Toko Hashizume

**Affiliations:** Department of Breast and Thyroid Surgery, Aizawa Hospital, 2-5-1 Honjo, Matsumoto, Nagano 390-8510, Japan; Department of Breast and Thyroid Surgery, Aizawa Hospital, 2-5-1 Honjo, Matsumoto, Nagano 390-8510, Japan; Department of Thoracic Surgery, Aizawa Hospital, 2-5-1 Honjo, Matsumoto, Nagano 390-8510, Japan; Department of Breast and Thyroid Surgery, Aizawa Hospital, 2-5-1 Honjo, Matsumoto, Nagano 390-8510, Japan

**Keywords:** intramammary lymph node, VATS, video-assisted thoracic surgery, breast cancer

## Abstract

The use of video-assisted thoracic surgery (VATS) has increased in recent years. We herein report a case wherein suspected intramammary lymph node (IM) recurrence of breast cancer was treated using the thoracic approach (VATS). A 53-year-old woman had undergone right total mastectomy, axillary lymph node dissection, and implant-based reconstruction for right breast cancer 19 years ago. Hormone therapy was commenced postoperatively. Positron emission tomography as health check up performed at another hospital 1 year prior to presentation revealed enlargement of the right IMs, suggesting recurrence of breast cancer. IM biopsy was performed using VATS to preserve the artificial breast implant. The operative time and blood loss were 157 min and 20 ml, respectively. The postoperative course was favourable. IM biopsy revealed reactive enlargement owing to inflammation. In conclusion, VATS is a safer approach that yields superior outcomes in terms of appearance care.

## Introduction

Postoperative intramammary lymph node (IM) oligo-recurrence of breast cancer, a very rare phenomenon, has been reported in 0.3% of cases, whereas IM recurrence following metastasis of other lesions has been reported in 1.0%–6.5% of cases [1, 2]. IM biopsy is often required following total mastectomy and artificial breast reconstruction to diagnose metastatic recurrence or inflammation when the IM on the affected side is enlarged. Percutaneous needle biopsy is not possible due to the presence of breast implant,

IM biopsy using the conventional approach (chest wall incision) may require removal of the artificial implants. The use of video-assisted thoracic surgery (VATS) for the management of other types of carcinomas has increased in recent years. This case report describes the case of patient with suspected IM recurrence of breast cancer treated using VATS.

## Case report

**Figure 1:**
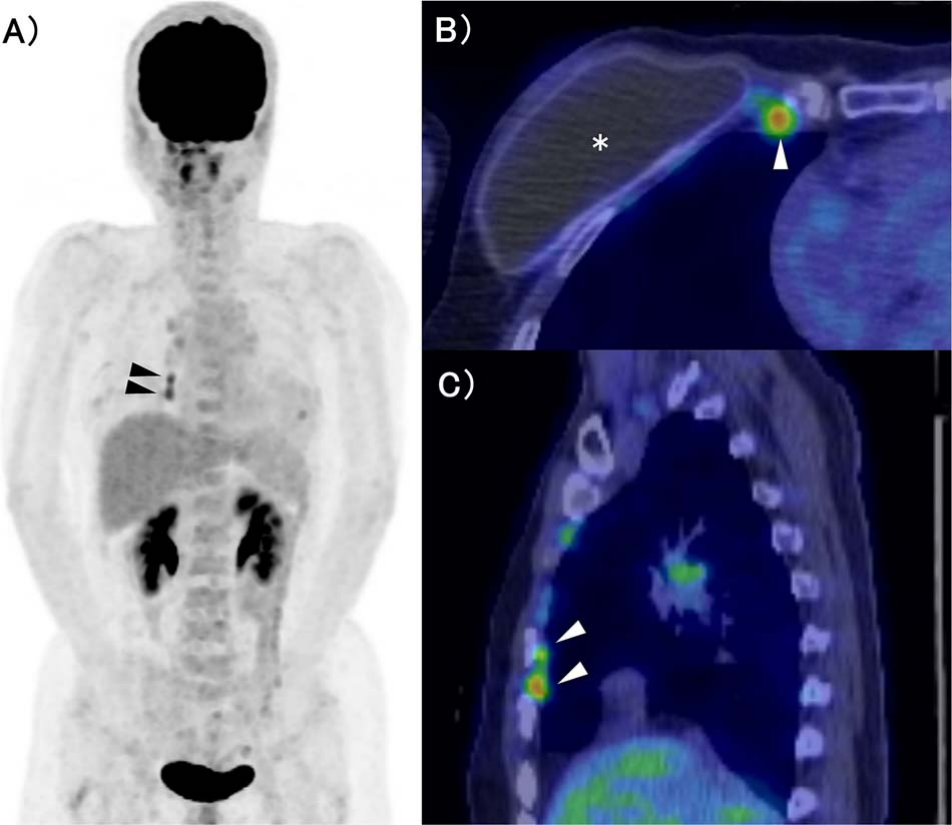
FDG-PET/CT. (A) MIP section. (B) Horizontal section. (C) Sagittal section. The asterisk and arrow indicate the artificial breast implant and IMs, respectively. FDG accumulation was observed in the IMs. FDG: Fluorodeoxyglucose, PET: Positron Emission Tomography, CT: Computed Tomography, MIP: Maximum Intensity Projection, IM: Internal Mammary lymph node.

**Figure 2:**
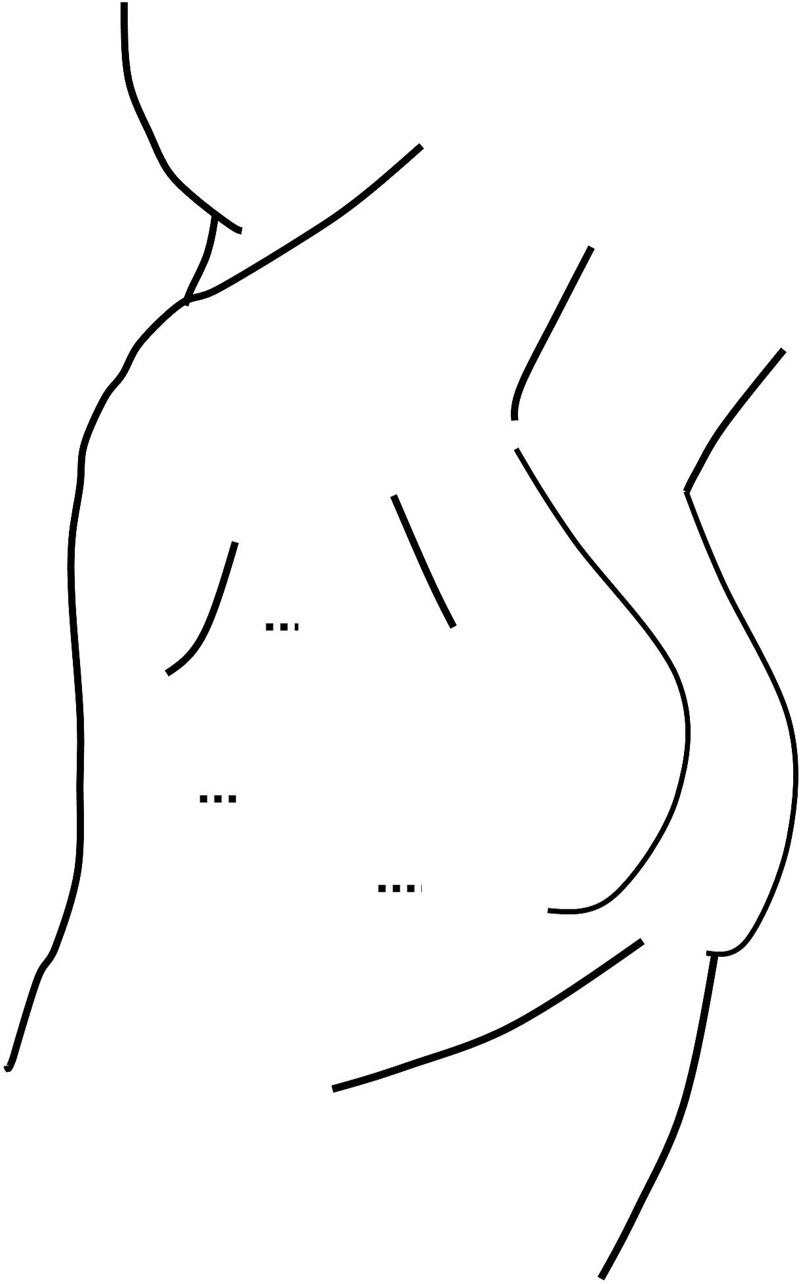
The surgery was performed in the left lateral recumbent position. A total of three incisions were made in the second, third, and fourth intercostal spaces. The dotted lines indicate the wounds.

**Figure 3:**
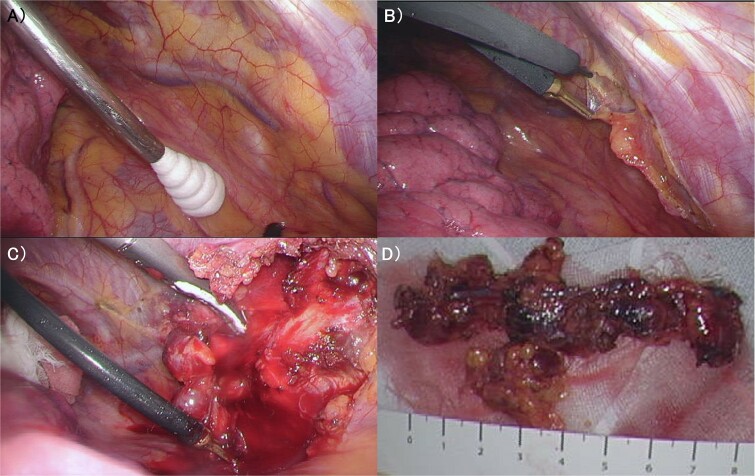
(A) The internal mammary artery and vein can be seen thorough the parietal pleura. (B) The parietal pleura were dissected along the internal mammary artery and vein. (C) En bloc resection of IM from the chest wall was performed. (D) The enlarged IM was widely resected. IM: Internal Mammary lymph node.

The patient was a 53-year-old woman who had undergone right total mastectomy, axillary lymph node dissection, and implant-based reconstruction for right breast cancer 19 years prior to presentation. The histological findings were TisN0M0, ductal carcinoma in situ (DCIS), and immunostaining was ER positive, PgR positive, and HER2 positive. Hormone therapy (luteinizing hormone-releasing hormone agonist for 2 years and tamoxifen for 6 years) was commenced postoperatively. Fluorodeoxyglucose (FDG) positron emission tomography/computed tomography as health check up performed at another hospital 1 year prior to presentation revealed enlargement of the right IMs, suggesting recurrence of breast cancer (Fig. 1). There were no particular findings to suspect capsular contracture and the Baker Grade was assumed to be grade I with no calcification and aesthetic status was quite good. IM biopsy was performed using VATS to preserve the artificial breast implant. The operative procedure was as follows; a total of three incisions were made in the second, third, and fourth intercostal spaces in the left lateral recumbent position (Fig. 2). The internal mammary artery and vein can be seen thorough the parietal pleura (Fig. 3A). The parietal pleura were dissected along the internal mammary artery and vein (Fig. 3B). En bloc resection of IM from the chest wall was performed (Fig. 3C). The enlarged IM was widely resected (Fig. 3D). The operative time and blood loss were 157 min and 20 ml, respectively. The postoperative course was favourable. The chest drainage tube was removed on postoperative day (POD) 1, and the patient was discharged at POD 3. IM revealed reactive enlargement caused by inflammation.

## Discussion

Sutton *et al*. reported that IM enlargement occurs in 37.6% of patients following silicone breast implant insertion, which is unexpectedly high [3]. Postoperative IM oligo-recurrence of breast cancer, a very rare phenomenon, has been reported in 0.3% of cases, whereas IM recurrence following metastasis of other lesions has been reported in 1.0%–6.5% of cases [1, 2].

Kawaguchi *et al*. also described two cases of enlarged IM resected by VATS that were pathologically diagnosed as inflammation [4].

Swollen IMs with FDG accumulation are often pathologically diagnosed as benign in patients who have undergone breast reconstruction. These cases accounted for 87.5% (7/8) of the cases in the study by Sutton *et al*. [3]. Thus, enlarged IMs should not be considered to indicate recurrence based on FDG accumulation.

The National Comprehensive Cancer Network (NCCN) guideline recommends radiation therapy for local recurrence of IM, with no mention of surgical resection [5].

Several studies have reported the use of VATS for IM biopsy, with operative time, blood loss, and length of hospital stay of 42.2–156 min, 41–65.5 ml, and a few days, respectively [4, 6–11]. Thus, IM biopsy using VATS may be a less invasive procedure than the conventional approach (chest wall incision). Moreover, compared with the conventional approach, the magnification effect associated with VATS increases visibility and safety.

Radiation is difficult to indicate, and surgical resection may be an optional treatment in cases wherein the patient has undergone artificial breast reconstruction or chest wall radiation. IM enlargement after breast reconstruction is relatively frequent; thus, IM biopsies using minimally invasive VATS can be used for diagnosis and treatment.

The greatest benefit of VATS is that resection of the artificial implant can be avoided in patients who have undergone breast implantation. The wound was small and located in the lateral thoracic region in our patient, making it less visible. Possible complications of VATS are iatrogenic pneumothorax and IM artery or vein injury, but the risk of lung injury was considered low if we take care of the initial incision for scope insertion. In addition, if bleeding occurs, haemostasis can be easily achieved by magnification effect.

Although there have been no previous reports, the use of single hole thoracoscopic or robotic surgery could provide an even less invasive procedure, but this is a future challenge.

Silicone lymphadenopathy can occur in the ipsilateral axillary lymph nodes and IM [12–15]. Middleton *et al*. reported that migration of free silicone particle can lead to the formation of silicone granulomas secondary to a foreign-body reaction; however, this was not revealed pathologically in our case, which remains unclear [14].

As the initial surgery was 19 years ago and the histology was DCIS, recurrence was unlikely and the possibility of lymphadenopathy due to breast implant was high. Close follow-up and repeat imaging evaluation may have been an option.

In conclusion, VATS is a safe approach for suspected IM metastases following total mastectomy and artificial implantation that yields superior outcomes in terms of appearance care although as for limitation, there is little evidence for IM biopsy and it is not mentioned in the NCCN guidelines. Further studies are expected.

## Data Availability

The data sets used in this study are available from the corresponding author upon request.
